# Blueberry polyphenols increase lifespan and thermotolerance in Caenorhabditis elegans

**DOI:** 10.1111/j.1474-9726.2006.00192.x

**Published:** 2006-02

**Authors:** Mark A Wilson, Barbara Shukitt-Hale, Wilhelmina Kalt, Donald K Ingram, James A Joseph, Catherine A Wolkow

**Affiliations:** 1 Laboratory of NeurosciencesNational Institute on Aging, Intramural Research Program, Baltimore, MD 21224, USA; 2 United States Department of AgricultureHuman Nutrition Research Center on Aging, Tufts University, Boston, MA 02111, USA; 3 Agriculture and Agri-Food Canada32 Main Street, Kentville, Nova Scotia, Canada; 4 Laboratory of Experimental GerontologyNational Institute on Aging, Intramural Research Program, Baltimore, MD 21224, USA

**Keywords:** Aging, blueberry, *Caenorhabditis elegans*, lifespan, proanthocyanidin, thermal stress

## Abstract

The beneficial effects of polyphenol compounds in fruits and vegetables are mainly extrapolated from *in vitro* studies or short-term dietary supplementation studies. Due to cost and duration, relatively little is known about whether dietary polyphenols are beneficial in whole animals, particularly with respect to aging. To address this question, we examined the effects of blueberry polyphenols on lifespan and aging of the nematode, *Caenorhabditis elegans*, a useful organism for such a study. We report that a complex mixture of blue-berry polyphenols increased lifespan and slowed aging-related declines in *C. elegans*. We also found that these benefits did not just reflect antioxidant activity in these compounds. For instance, blueberry treatment increased survival during acute heat stress, but was not protective against acute oxidative stress. The blueberry extract consists of three major fractions that all contain antioxidant activity. However, only one fraction, enriched in proanthocyanidin compounds, increased *C. elegans* lifespan and thermotolerance. To further determine how polyphenols prolonged *C. elegans* lifespan, we analyzed the genetic requirements for these effects. Prolonged lifespan from this treatment required the presence of a CaMKII pathway that mediates osmotic stress resistance, though not other pathways that affect stress resistance and longevity. In conclusion, polyphenolic compounds in blueberries had robust and reproducible benefits during aging that were separable from antioxidant effects.

## Introduction

Plants synthesize an array of chemical compounds that are not involved in their primary metabolism. These ‘secondary compounds’ instead serve a variety of ecological functions, ultimately to enhance the plant's survival during stress (Winkel-Shirley, 2002). In addition, these compounds may be responsible for the beneficial effects of fruits and vegetables on an array of health-related measures (Liu, 2003). Previous research suggested that the combination of antioxidant/anti-inflammatory polyphenol compounds found in fruits and vegetables may show efficacy in reversing aging (Joseph *et al*., 2005). In particular, 8-week dietary supplementation with spinach, strawberry or blueberry (BB) extracts was effective in reversing age-related deficits in neuronal function and behavior in aged (19 month) F344 rats (Joseph *et al*., 1999). However, only the BB-supplemented group exhibited improved performance on tests of motor function. Specifically, the BB-supplemented group displayed improved performance on two motor tests that rely on balance and coordination, rod walking and the accelerating rotarod, while none of the other supplemented groups differed from controls on these tasks. A subsequent study using a BB-supplemented diet replicated these findings (Youdim *et al*., 2000). Results from both studies indicated that the significant effects of BB on behavior were due to a multiplicity of actions, in addition to those involving antioxidant and anti-inflammatory activity.

To further investigate the effects of blueberries on parameters related to aging, we designed a study to determine whether BB polyphenols can delay aging and prolong lifespan in a whole organism. For these studies, we required an organism with relatively short lifespan that could be assayed reproducibly and robustly, and for which the genetic and environmental factors affecting lifespan were well defined. The experimental organism that could best accommodate these requirements was the nematode, *Caenorhabditis elegans*, which has become a popular model for studying aging and longevity, due to its short 2- to 3-week lifespan, rapid generation time and experimental flexibility (Guarente & Kenyon, 2000).

Studies have shown that specific genetic and environmental factors influence aging and lifespan of *C. elegans* (Gems & Riddle, 2000; Garigan *et al*., 2002; Herndon *et al*., 2002). In addition, aspects of aging are similar between nematodes and mammals, including humans. For instance, sarcopenia, the loss of muscle mass, is a major feature of aging in humans and *C. elegans* and contributes to aging-related behavioral declines in both (Herndon *et al*., 2002; Glenn *et al*., 2004). Second, calorie restriction, the only known intervention that successfully extends lifespan in mammals, can prolong *C. elegans* lifespan (Lakowski & Hekimi, 1998). Finally, oxidative stress appears to be a major factor limiting lifespan in both *C. elegans* and humans (Larsen, 1993; Finkel & Holbrook, 2000). These findings show that studies of aging in *C. elegans* provide useful stepping stones for identifying genes and compounds that can prolong lifespan in humans.

Here, we report that treatment with total BB polyphenols, or a proanthocyanidin (PAC)-enriched fraction from BB, could prolong adult lifespan and delay aging in *C. elegans*. These treatments increased thermotolerance, but did not improve resistance to oxidative stress. BB treatment was correlated with reduced basal levels of *hsp* mRNA, indicating these compounds had direct or indirect effects on gene expression. Our genetic analysis indicated that BB polyphenols may act through a CaMKII signaling pathway to affect *C. elegans* lifespan. These findings show that natural compounds from blueberries can provide antiaging benefits *in vivo* in an intact organism.

## Results

### Blueberry polyphenols extend *C. elegans* lifespan

**Fig. 1:**
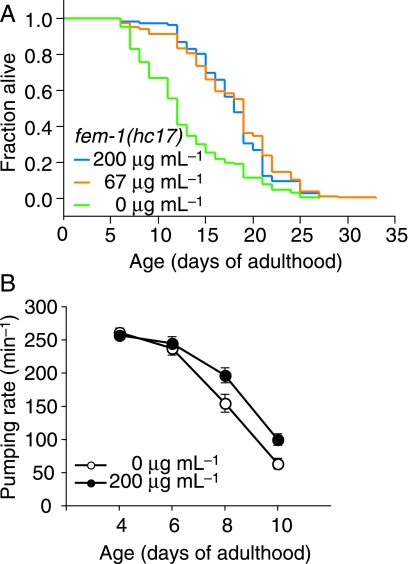
Blueberry polyphenols extend lifespan and slow aging in *Caenorhabditis elegans*. (A) Treatment with blueberry polyphenols [67 µg mL^−1^ (orange) or 200 µg mL^−1^ (blue)] extended mean lifespan in *fem-1(hc17)* animals grown at 25 °C (untreated control, green). (B) Blueberry polyphenols slowed the decline in pharynx pumping during aging. Open circles, untreated; filled circles, treated with 200 µg mL^−1^ blueberry polyphenols. Average pumping rate (pumps per minute) in 14 animals scored in two trials; error bars indicate SEM among individual animals scored; *t*-test, untreated vs. treated, day 8, *P* = 0.023, day 10, *P* = 0.004.

Adult wild-type animals grown under our standard laboratory conditions at 25 °C have a mean lifespan of 12.7 days and average maximum lifespan of 19.7 days. On media containing either crude BB extract (*Vaccinium angustifolium*) or a C18 column fraction containing their bulk polyphenols, mean lifespan of wild-type animals was lengthened by 28% (Fig. 1A, Table 1). Maximum lifespan was also increased by an average of 14% in all trials (*P =* 0.007; 0 µg mL^−1^, *n* = 17 trials; 200 µg mL^−1^, *n* = 16 trials). Similar results were obtained using a crude extract of a different species of blueberry (*Vaccinium asheii*) (not shown).

**Table 1:** The effect of genetic and environmental variables upon lifespan in the presence of blueberry polyphenols

		Mean lifespan, SEM (*n*)		
			
Genotype	Treatment	Control	Treated	Change
*fem-1(hc17)*	BB crude	12.1, 0.63 (73)	16.6, 0.46 (46)	1.37*
‘’	BB 25 °C	12.7, 0.23 (362)	16.2, 0.23 (350)	1.28**
‘’	BB 20 °C	16.1, 0.26 (237)	18.7, 0.26 (210)	1.16**
‘’	BB 15 °C	22.9, 0.74 (44)	22.2, 0.92 (38)	0.97
‘’	amp	13.1, 0.66 (60)	17.5, 0.56 (66)	1.34**
‘’	amp + BB	13.1, 0.66 (60)	18.1, 0.46 (70)	1.38**
*mev-1(kn1)*	BB	9.1, 0.28 (101)	8.6, 0.28 (98)	0.95
*sir-2(ok434)*	BB	12.2, 0.54 (76)	15.9, 0.58 (68)	1.30**
*osr-1(rm1)*	BB	16.3, 0.31 (151)	15.7, 0.37 (78)	0.96
*unc-43(n1186)*	BB	13.1, 0.31 (108)	12.8, 0.48 (61)	0.98
*sek-1(ag1)*	BB	11.7, 0.73 (33)	10.9, 0.64 (26)	0.93
*daf-16(mgDf50)*	BB	10.2, 0.38 (53)	12.3, 0.34 (42)	1.21**
‘’	amp	11.5, 0.38 (53)	12.7, 0.38 (53)	1.10*
‘’	amp + BB	11.5, 0.38 (53)	14.7 0.38 (53)	1.28**
*skn-1(zu67)*	BB	9.9, 0.52 (36)	13.1, 0.54 (43)	1.32**

* *P* ≤ 0.05,

** *P* ≤ 0.001, log-rank. Aging assays performed at 25 °C, unless noted, except *mev-1(kn1)* which was carried out at 25 °C and 20 °C, with or without FUDR, with similar results. Treatments: crude at 1.5 mg mL^−1^; BB, BB polyphenols at 200 µg mL^−1^; amp, ampicillin at 100 µg mL^−1^.

Lifespan in *C. elegans* is affected by temperature (Gems *et al*., 1998). Animals grown at 25 °C have shorter adult lifespan than animals grown at 15 °C, although both temperatures are considered within the normal range for *C. elegans*. The effects of BB polyphenols on *C. elegans* lifespan were also temperature dependent. BB treatment prolonged lifespan in animals grown at 25 °C or 20 °C, but no significant benefits were observed upon lifespan at 15 °C (Table 1).

By several measures, BB treatment slowed aging in *C. elegans*, rather than simply improving survival at old age. One measure of aging in *C. elegans* is the speed of pharynx contraction, or pumping (Bolanowski *et al*., 1981; Huang *et al*., 2004). Young adult animals pump 250–300 times per minute and pumping declines gradually with increasing age. BB treatment was associated with higher pumping rates at adult days 8 and 10 (Fig. 1B).

**Fig. 2:**
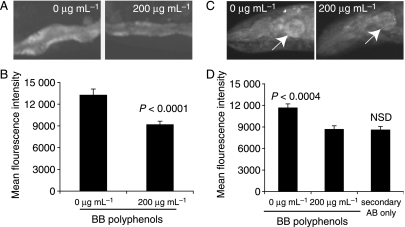
Markers of aging and oxidative damage are reduced in animals treated with BB polyphenols. (A)Intestinal autofluorescence from lipofuscin in representative day 16 animals with 0 µg mL^−1^ (left) or 200 µg mL^−1^ blueberry polyphenols (right). (B)Mean fluorescence intensity from intestinal lipofuscin in day 16 adults treated with indicated amounts of blueberry polyphenols; 0 µg mL^−1^, *n* = 24 animals; 200 µg mL^−1^, *n* = 26 animals. (C)Representative images of pharynxes from BB-treated or control animals on adult day 14 immunostained with antisera specific for 4-HNE. Arrows designate terminal bulb. Similar results were obtained for 4-HNE immunofluorescence in the somatic gonad. (D) Mean fluorescence intensity of 4-HNE immunofluorescence in pharynx terminal bulbs from day 14 adults. Third bar shows background fluorescence measured in animals stained with secondary antibody only. *P*-values are *t*-test vs. 200 µg mL^−1^; NSD, no significant difference; *n* = 18 animals (0 µg mL^−1^); 16 animals (200 µg mL^−1^); *n* = 4 animals (secondary antibody control).

BB treatment also delayed the accumulation of aging-related cellular damage. One marker for cellular damage during aging is the intracellular level of lipofuscin, autofluorescent material that accumulates in aging cells. (Brunk & Terman, 2002). Lipofuscin levels increase with aging in many organisms, including *C. elegans* (Hosokawa *et al*., 1994). We examined lipofuscin levels in the intestines of control and BB-treated adult day 16 animals. Consistent with the expectation that BB polyphenols could slow aging, intestinal lipofuscin levels were reduced 20% in BB-treated animals, compared with controls (Fig. 2A,B). 4-Hydroxynonenol (4-HNE) is a lipid peroxidation product that accumulates with aging in many animals (Chiarpotto *et al*., 1995). In *C. elegans*, a correlation between 4-HNE levels and aging has not yet been demonstrated, although levels of 4-HNE-modified protein have been correlated with lifespan (Ayyadevara *et al*., 2005). BB treatment also reduced levels of 4-HNE in 14-day adults (Fig. 2C,D).

### BB treatment delayed aging-related increase in heat-shock protein mRNA levels

**Fig. 3:**
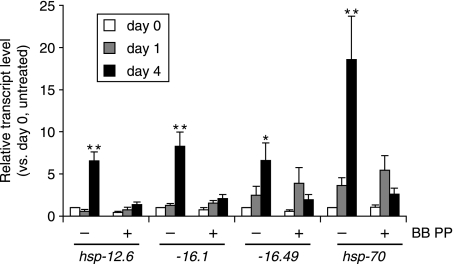
BB polyphenols reduced aging-related increase of inducible *hsp* transcripts. Expression levels of small heat-shock proteins, relative to actin, were determined by RT-PCR in populations treated with 200 g/ml of blueberry polyphenol (BB PP) at 25 °C. Graph shows mean of two independent experiments with SEM; **P < 0.01 vs. day 0 within treatment; *P < 0.05 vs. day 0 within treatment.

We next examined the effects of BB treatment on a transcriptional marker of aging. Several independent analyses of gene expression during aging in *C. elegans* have revealed that heat-shock protein mRNA levels increase with age (Lund *et al*., 2002; Golden & Melov, 2004). Using real-time PCR, we confirmed that expression of *hsp-12.6, -16.1, -16.49* and *-70* increased markedly in control animals between days 0 and 4 of adulthood under our laboratory conditions (Fig. 3). BB treatment blocked this increase and mRNA levels for these *hsp*s remained at basal levels through to adult day 4. From this experiment, we conclude that BB polyphenols delayed changes in *hsp* expression that are normally associated with aging in *C. elegans*, consistent with the observation that BB treatment delayed morphological features of aging.

### Proanthocyanidin components of blueberries enhance longevity

**Fig. 4:**
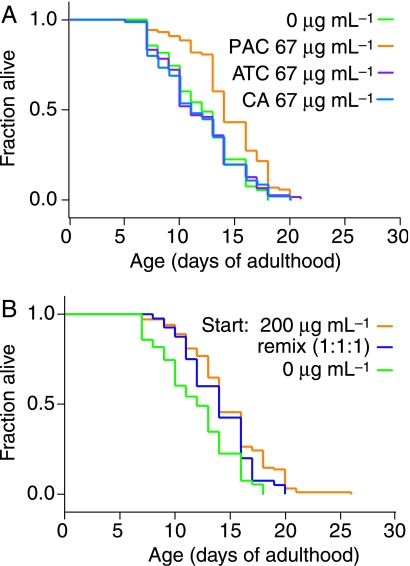
A PAC-enriched fraction of BB contained components sufficient to extend *Caenorhabditis elegans* lifespan. The total blueberry polyphenols were fractionated by C18 and Sephadex LH20 to produce fractions enriched in anthocyanins (ATC), proanthocyanidins (PAC) or chlorogenic acid (CA). Purchased, purified CA was used for CA experiments. Each fraction was assayed for effects on *C. elegans* longevity at a concentration of 67 µg mL^−1^, similar to that in the complete extract. (A)Only the PAC-enriched fraction (orange) prolonged lifespan. No longevity benefits were observed for the ATC-enriched fraction (purple) or purified CA (blue); 0 µg mL^−1^, *n* = 97 animals, 2 trials; ATC, *n* = 99, 2; CA, *n* = 91, 2; PAC, *n* = 88, 2. Complete statistics for PAC trials are presented in Table 2. (B) The reconstituted extract, produced by mixing equal mass ratios of fractions in (A) also conferred the same lifespan extension as the starting mix of polyphenols (remix, *n* = 40 animals, 1 trial; start, *n* = 98, 2).

Blueberries contain a mixture of different polyphenol compounds that can be separated into three primary fractions enriched in either anthocyanins (ATC), proanthocyanidins (PAC) or hydroxycinnamic esters, mainly chlorogenic acid (CA). Major components of each of these fractions have been shown to confer significant antioxidant activity and ATC can protect cells against oxidative stress *in vitro* (Youdim *et al*., 2000; Zheng & Wang, 2003). To determine which fraction(s) delayed aging, we assayed their effects on *C. elegans* lifespan. Neither the ATC-enriched fraction nor purified CA had any significant effect on longevity (Fig. 4A) (control, 12.0 ± 0.34 days; ATC, 11.7 ± 0.36 days, *P* = 0.96 vs. control; CA, 11.7 ± 0.39 days, *P* = 0.99). However, treatment with the PAC-enriched fraction increased lifespan to a similar extent as the starting BB polyphenol mixture or the remixed fractions (Fig. 4A,B) (PAC, 14.4 ± 0.36 days, *P* < 0.0001 vs. control; start, 14.8 ± 0.36 days, *P* < 0.0001; remix, 14.0 ± 0.48 days, *P* < 0.0001; complete statistics for lifespan trials with PAC-enriched fraction are presented in Table 2). Thus, components of the PAC-enriched fraction of blueberries could extend lifespan in *C. elegans*.

**Table 2:** Effect of PAC-enriched fraction of BB polyphenols on *fem-1(hc17)* adult lifespan at 25 °C, results for individual and combined trials

Trial	Treatment	Adult lifespan, 25 °C (mean, SE)	(*n*)	*P* vs. control (log-rank)
1	Control	12.2 days, 0.47	(40)	
	PAC 67 µg mL^−1^	13.4 days, 0.66	(29)	0.063
	PAC 200 µg mL^−1^	13.2 days, 0.53	(33)	0.171
2	Control	11.8 days, 0.47	(57)	
	PAC 67 µg mL^−1^	14.8 days, 0.42	(59)	< 0.0001
	PAC 200 µg mL^−1^	14.0 days, 0.43	(59)	0.0026
1 + 2	Control	12.0 days, 0.34	(97)	
	PAC 67 µg mL^−1^	14.4 days, 0.36	(88)	< 0.0001
	PAC 200 µg mL^−1^	13.7 days, 0.34	(92)	0.0008

### Effects of blueberry polyphenols on intrinsic stress resistance

**Fig. 5:**
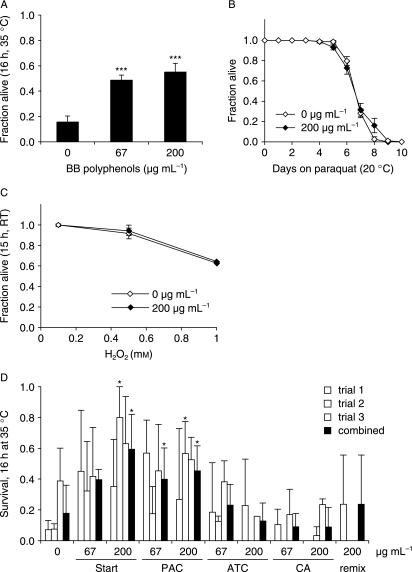
Treatment with blueberry polyphenols improved thermotolerance, but not oxidative stress resistance. (A)Fractional survival at 35 °C for day 5 *fem-1(hc17)* or animals with indicated treatments (0, 67 µg mL^−1^ or 200 µg mL^−1^ BB). Shown is average survival in 3–5 experiments with 30–60 animals/experiment; error bars, SEM; total number of animals tested: 173 (0 µg mL^−1^), 208 (67 µg mL^−1^), 117 (200 µg mL^−1^); ***P < 0.0001, *t*-test. (B) Survival on 10 mm paraquat was not affected by BB polyphenols; *n* = 74–91 animals. (C) Survival on hydrogen peroxide was not affected by 200 µg mL^−1^ BB polyphenols; error bars, SEM; *n* = 59–98 animals in three trials. (D) Effect of BB polyphenol subfractions on thermotolerance in *fem-1(hc17)* animals. Sixteen-hour (16-h) survival at 35 °C was assessed for animals treated with BB polyphenol subfractions shown in Fig. 4, or untreated control animals, as described in Experimental procedures; *n*≥ 25 animals/trial; *P ≤ 0.05, *t*-test, treated vs. untreated controls.

One possible explanation for the beneficial effects of BB polyphenols on aging in *C. elegans* is that these compounds were able to increase cellular stress resistance. In several studies, increased longevity was closely associated with improved survival under conditions of heat or oxidative stress (Lithgow *et al*., 1995; Muñoz & Riddle, 2003). To test this possibility, we examined stress resistance of BB-treated animals. Thermotolerance was increased significantly by BB treatment. In wild-type animals, treatment with BB polyphenols was correlated with a 2.5-fold increase in 16-h survival at 35 °C (Fig. 5A). Lifespan and thermotolerance were linked, as thermotolerance was increased by treatment with the PAC-enriched fraction, but not the ATC-enriched fraction nor CA (Fig. 5D).

Interestingly, BB treatment did not improve survival under mild to severe oxidative stress. Resistance to oxidative stress was examined by exposing animals to hydrogen peroxide or paraquat, an intracellular free-radical-generating compound. BB treatment was not associated with any increase in survival in the presence of hydrogen peroxide or paraquat (Fig. 5B,C). To further examine whether BB polyphenols could protect against acute oxidative stress, we examined *mev-1(kn1)* animals, which have a mutation in the cytochrome *b* large subunit of mitochondrial complex II (Ishii *et al*., 1998). This mutation results in overproduction of superoxide and increased oxidative stress, along with accelerated aging and reduced lifespan (Hosokawa *et al*., 1994; Senoo-Matsuda *et al*., 2001). Consistent with the finding that BB did not protect against extrinsic oxidative stress, BB treatment also did not significantly affect lifespan of *mev-1(kn1)* animals (Table 1).

### Effects of BB treatment on expression of stress-inducible genes

We next considered the possibility that BB treatment acted as a mild stressor that induced expression of protective enzymes and this increased gene expression delayed aging (Lithgow *et al*., 1995). To investigate this possibility, we examined the effects of BB treatment on the expression of several stress-inducible genes. Although BB treatment improved thermotolerance, the mRNA levels for a number of stress-inducible genes were not altered by BB treatment in any consistent way (Supplementary Table S1). In this analysis, we were careful to select stress-inducible genes regulated by several different pathways. In addition, the earlier experiment showed that mRNA levels of inducible *hsp*s were not increased in BB-treated animals (Fig. 3), nor was BB treatment associated with consistently greater *hsp* mRNA induction following heat-shock compared with untreated controls (Supplementary Table S2). Together, these gene expression analyses show that BB treatment did not cause a general induction of stress-response genes under normal growth conditions, but appeared to increase overall health, which may have promoted greater survival under thermal stress. However, we cannot rule out the possibility that BB polyphenol treatment may increase expression levels of stress-response genes during old age.

### Blueberry polyphenols do not appear to prolong lifespan through antimicrobial effects

**Fig. 6:**
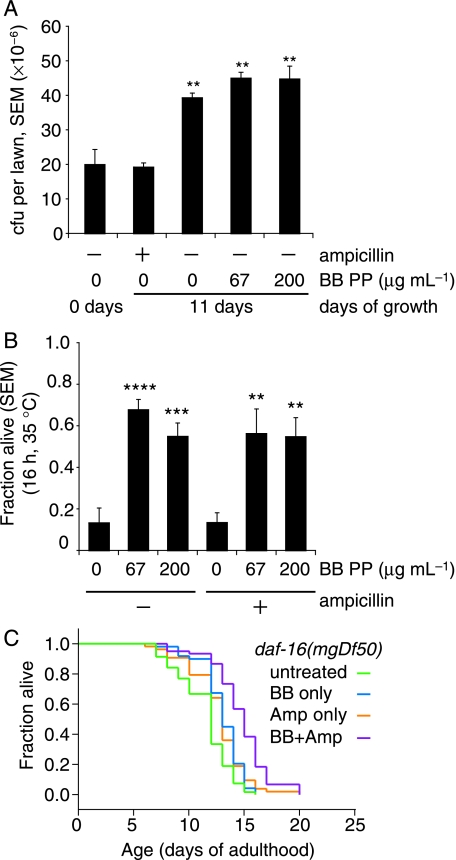
BB and antimicrobial treatment had different effects on *Caenorhabditis elegans* thermotolerance and lifespan. (A)BB treatment did not inhibit bacterial growth in aging assays. Bacterial lawn growth was monitored over 11 days under conditions replicating those of *C. elegans* aging assays. Shown are the average number of colony forming units (cfu) per lawn after indicated treatments in two trials, **P < 0.01. (B) Effects of blueberry polyphenols and ampicillin on *C. elegans* thermotolerance measured as survivorship at 35 °C after 16 h, as in Fig. 5(A). Ampicillin treatment alone had no effect on thermotolerance. Shown are average survivorships in three trials, with at least 20 animals per trial, **P < 0.01, ***P < 0.001, ****P < 0.0001. (C) BB polyphenols (200 µg mL^−1^) and ampicillin had additive effects on *daf-16(mgDf50)* lifespan at 25 °C (purple curve). *daf-16(mgDf50)*, untreated control, mean 11.6 days, 0.28 SE, *n* = 69; 200 µg mL^−1^ BB polyphenols, mean 13.0 days, 0.27 SE, *n* = 49; 100 µg mL^−1^ ampicillin, mean 12.7 days, 0.35 SE, *n* = 53; ampicillin + BB polyphenols, mean 14.7 days, 0.33 SE, *n* = 60; *P* < 0.0001, log-rank, for Amp. vs. BB+Amp and for BB vs. BB+Amp.

One contributor to late-age mortality in *C. elegans* is the detrimental effect of the bacterial food source (Gems & Riddle, 2000; Garigan *et al*., 2002). Accordingly, *C. elegans* lifespan could be increased approximately 30% when bacterial growth was arrested by ampicillin (Table 1). We observed that lifespan of BB-treated wild-type animals was not further extended by the presence of ampicillin to arrest bacterial growth (Table 1), indicating that BB may have extended lifespan by relieving microbial stress. To investigate this possibility, we first tested whether the BB polyphenols that prolong lifespan inhibited bacterial growth. We monitored growth of the bacterial lawn under conditions identical to *C. elegans* aging assays, 11 days on NGM agar at 25 °C, in the presence or absence of BB. During this period, untreated bacterial lawns completed approximately one population doubling, while bacterial growth was arrested after ampicillin treatment. In contrast, BB polyphenols had no effect on bacterial population growth at the doses tested in lifespan assays (Fig. 6A). Furthermore, *C. elegans* nematodes grown on ampicillin-treated bacteria displayed no increase in thermotolerance, nor did the combination of ampicillin and BB together enhance thermotolerance (Fig. 6B). Together these findings show that ampicillin and BB polyphenols had distinct effects during aging, supporting the conclusion that BB polyphenols do not extend *C. elegans* longevity through simple antimicrobial effects.

### Genetic requirements for increased survival from BB treatment

Pathways for induction of stress-response genes that affect lifespan have been identified in *C. elegans*. Because BB treatment might improve survival by acting through these genes, we examined whether mutations in the four major stress response and longevity pathways impaired the ability of BB to prolong lifespan. The premise of these experiments was that BB treatment would not extend lifespan in mutant animals missing a gene required for BB's beneficial effects.

Treatment with the polyphenol, resveratrol, or related compounds, can increase *C. elegans* lifespan through *sir-2.1*, which encodes a histone deacetylase-like protein that integrates metabolic status with lifespan (Tissenbaum & Guarente, 2001; Wood *et al*., 2004). BB treatment extended lifespan of *sir-2.1(ok434)* animals which lacked *sir-2.1* gene activity, showing that BB polyphenols and resveratrol did not act through the same mechanism to increase *C. elegans* lifespan (Table 1).

**Fig. 7:**
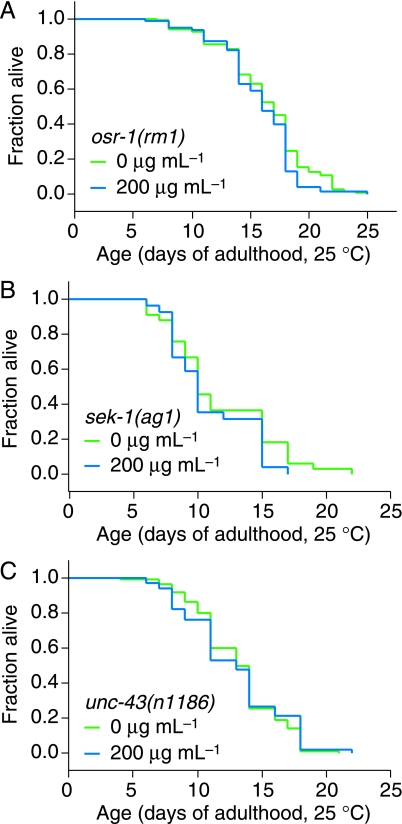
Increased lifespan from blueberry polyphenol treatment is blocked by mutations in the osmotic stress resistance pathway. Lifespan in *osr-1(rm1)* (A), *sek-1(ag1)* (B), and *unc-43(n1186)* (C) was not lengthened by treatment with 200 µg mL^−1^ blueberry polyphenols. Lifespan survival statistics are contained in Table 1.

Survival in hyperosmotic environments requires the activity of a novel protein, OSR-1, which couples to SEK-1/MAPKK through UNC-43/CaMKII (Solomon *et al*., 2004). SEK-1/MAPKK is also required for resistance to pathogenic bacteria and oxidative stress (Kim *et al*., 2002; Kondo *et al*., 2005). BB treatment did not prolong lifespan of *osr-1(rm1)* animals, suggesting that BB polyphenols might act through *osr-1* (Fig. 7A, Table 1). Similarly, BB treatment did not affect lifespan of *sek-1(ag1*) or *unc-43(n1186)* animals (Fig. 7B,C, Table 1). These results implicate the OSR-1/UNC-43/SEK-1 pathway as a target for BB polyphenols in *C. elegans*.

In *C. elegans*, two transcription factors, DAF-16 and SKN-1, promote expression of antioxidant or detoxification enzymes. The DAF-16/FOXO transcription factor promotes expression of genes that confer extended longevity and enhanced stress resistance (Lee *et al*. 2003; Murphy *et al*. 2003). SKN-1, which is related to vertebrate Nrf proteins, promotes expression of detoxification enzymes in response to oxidative stress (An & Blackwell, 2003). BB treatment prolonged lifespan of both *daf-16(mgDf50)* and *skn-1(zu67)* animals, showing that BB may act independently of these genes (Table 1, Supplementary Fig. S1A). BB treatment also significantly improved thermotolerance in *daf-16(mgDf50)* animals (Supplementary Fig. S1B). Interestingly, BB and ampicillin had additive effects on *daf-16(mgDf50)* lifespan, further supporting the conclusion that BB polyphenols do not act through simple antimicrobial effects (Fig. 6C).

## Discussion

Dietary consumption of compounds in blueberries and other fruits and vegetables can attenuate age-related declines in several physiological and functional indices (Joseph *et al*., 2005). Using the short-lived nematode, *C. elegans*, we have established a genetic system to examine the effects of BB polyphenols upon longevity and aging. This work shows that treatment with BB polyphenols, or a PAC-enriched fraction alone, produced moderate extensions of both the mean and average maximum adult lifespan as well as improved thermotolerance. While this effect was modest as compared with mutations in longevity genes, it was significant, reliable and robust (Kenyon *et al*., 1993). Finally, we showed that the beneficial effects of BB treatment appear to require the activity of the *osr-1* pathway that also governs osmotic stress resistance.

By using *C. elegans* for these experiments, a genetic analysis of the beneficial effects of BB was possible. We showed that BB could not protect animals against the elevated oxidative stress imposed by the *mev-1(kn1)* mutation, consistent with our finding that BB also did not protect wild-type animals against oxidative stress from paraquat or hydrogen peroxide treatments. In addition, we found that three stress-response pathways were dispensable for the beneficial effects of BB polyphenols upon lifespan. These pathways were represented by: (i) *sir-2.1*, which promotes longevity during calorie restriction, (ii) *daf-16*, which promotes longevity and stress resistance, and (iii) *skn-1*, which promotes oxidative stress resistance. Specifically, BB treatment was able to extend lifespan of animals lacking any of these three genes. In contrast, BB treatment did not prolong lifespan of animals with defects in the *osr-1*/*unc-43*/*sek-1* pathway that promotes resistance to osmotic stress. One interpretation of this latter finding is that the effects of BB treatment are mediated, at least in part, through these genes. Alternatively, mutations in the *osr-1* pathway could place animals under some stress that is not affected by BB treatment. To date, *osr-1* pathway mutants have not been thought to suffer from different causes of death than wild-type animals, suggesting that the latter explanation is less likely. Therefore, we propose that BB exerts beneficial effects through interactions with the *osr-1* pathway.

Fractionation of the bulk polyphenols showed that BB's benefits on longevity and thermotolerance cofractionated with the PAC-enriched fraction, and not with ATC or CA, two other major constituents of blueberries that have antioxidant activity *in vitro*. These findings may either show that (i) the antioxidant effects of these compounds *in vitro* are not relevant to their *in vivo* effects in this model, or (ii) differential bioavailability of these compounds is a major determinant of their effects in whole animals. Due to the structural diversity in these fractions, we did not attempt to directly measure the levels of polyphenols inside the animals. However, we did observe the accumulation of BB pigments (primarily ATC) inside the intestines of treated animals, leading us to conclude that components of BB extracts could enter the body of these animals. Our analysis showed that BB did not protect animals against oxidative stress from paraquat, hydrogen peroxide or genetic mutation [*mev-1(kn1)*], which increases superoxide production *in vivo* (Senoo-Matsuda *et al*., 2001). These findings are consistent with the hypothesis that *in vitro* antioxidant activities of these compounds are not necessarily strong predictors of their benefits *in vivo* in whole animals.

As a part of this study, we examined possible mechanisms for the beneficial effects of BB treatment. As mentioned, BB treatment was not correlated with increased oxidative stress resistance, nor did BB appear to act solely by relieving microbial stress. One reasonable hypothesis is that BB's antioxidant activity was only sufficient to cope with low-level oxidative stress, such as induced by thermal stress or during aging. Alternatively, BB PACs might have other activities that protected cells specifically during thermal stress.

Given this evidence, we propose the following model for BB's effects on *C. elegans* lifespan. BB appears to protect animals against some aging-associated stress that overlaps with the stress imposed by high temperature. One possibility is that BB polyphenols protect cells from low levels of free radicals. Alternatively, BB polyphenols might alter the activity of signaling pathways required for response to thermal stress. Given the beneficial effect of BB on lifespan and thermotolerance, it was somewhat unexpected that the *osr-1/unc-43/sek-1* pathway for osmotic stress resistance was required for these effects of BB polyphenols, especially since *osr-1 has* not been found to affect thermotolerance (Solomon *et al*., 2004). One possibility is that BB and *osr-1* differentially affect outputs that can increase resistance to several types of stress. Further investigation of the cellular effects of BB treatment may reveal the nature of this genetic interaction.

Regardless of the specific mechanism involved, it is clear from these experiments that natural compounds available in blueberries can prolong lifespan of a whole organism, under certain conditions. This is a significant finding that lends support to previous experiments on cultured cells or short-term rodent studies showing beneficial effects in aging-related declines and stress resistance. Other studies have also shown that some other naturally available compounds can also prolong *C. elegans* lifespan under laboratory conditions (Harrington & Harley, 1988; Adachi & Ishii, 2000; Wu *et al*., 2002; Wood *et al*., 2004). With the exception of resveratrol, which acts through *sir-2.1*, the genetic requirements for these effects have not been examined (Wood *et al*., 2004). This work thoroughly examined *in vivo* the beneficial effects of compounds found in blueberries, and thus represents a significant advance in the study of the biological effects of natural compounds.

## Experimental procedures

### Strains and growth conditions

All strains were maintained at 15 °C on nematode growth medium (NGM) as described (Brenner, 1974). Strains used in this study were: N2, bristol (wild-type); BA17, *fem-1(hc17)*; GR1307, *daf-16(mgDf50)*; TK22, *mev-1(kn1)*; VC199, *sir-2.1(ok434)*; EU1, *skn-1(zu67)/nT1*; AM1, *osr-1(rm1)*; AU1, *sek-1(ag1)*; and MT2605, *unc-43(n498n1186)*. Before analysis, the *sir-2.1(ok434)* strain was backcrossed twice against wild type.

### Blueberry extracts and fractionation

Commercially prepared single strength wild blueberry juice (*Vaccinium angustifolium*) was applied to a preconditioned C18 column (Waters Canada Ltd, Mississauga, ON, Canada). The C18 column was washed with water to remove fructose, glucose and organic acids, which are abundant in blueberries, then with 100% methanol to obtain the total polyphenolic fraction. Methanol was removed under vacuum using a rotary evaporator (Buchi, Essen, Germany) at 30 °C. To obtain the proanthocyanidin fraction, the total polyphenol fraction was dissolved in 50% ethanol and applied to a column of Sephadex LH20 (Sigma-Aldrich, St. Louis, MO, USA), preconditioned with 50% ethanol. The column was washed with 60% ethanol until the eluant was colorless, and then with 70% aqueous acetone to elute the blueberry proanthocyanidins. To obtain the anthocyanin fraction, the total polyphenol fraction was dissolved in acidified water and washed four times with three volumes of ethyl acetate. Anthocyanins were partitioned into the water fraction, which was subsequently freeze dried. Pure chlorogenic acid (Sigma Chemical Company) was used instead of blueberry chlorogenic acid since the chlorogenic acid fraction obtained from blueberry fractionation is contaminated with minor flavonol, and flavonol glycoside components. These components are all contained in the ethyl acetate fraction obtained during anthocyanin isolation.

### Phenotypic assays

For aging assays, synchronous populations were obtained by allowing 5–10 hermaphrodites to lay eggs for 4 h to overnight. For ease of analysis, we used the adult sterile strain, *fem-1(hc17)*, as the wild-type strain to avoid progeny overgrowth in lifespan assays. Therefore, eggs were shifted to 25 °C, the nonpermissive temperature for fertility of *fem-1(hc17)*. Lifespan scoring was initiated after hermaphrodites completed the final larval molt, on the first day of adulthood. For aging assays with BB extracts, treatments were added to NGM agar plates on the first day of the lifespan assay. For lifespan assays with fertile strains, hermaphrodites were transferred daily for the first 4 days of adulthood to avoid progeny overgrowth. In these cases, all treatment plates were prepared on day 0 of adulthood. Statistical analyses and survival plots of lifespan data were performed with JMP analysis software (SAS Institute Inc, Cary, NC, USA). Pharynx pumping rates were scored on adults at room temperature (24 °C) under a Nikon SMZ1500 stereomicroscope (Nikon, Melville, NY, USA).

Thermotolerance assays were performed with hermaphrodites on adult day 5, after the majority of egg-laying had ceased. Animals were transferred onto 3-cm NGM agar plates supplemented as indicated and then incubated at 35 °C for 16 h. Survival was scored as the number of animals responsive to gentle touch as a fraction of the original number of animals on the plate. Animals that had died from dessication on the sides of the plate were censored. Paraquat-induced oxidative stress assays were performed with *fem-1(hc17)* hermaphrodites at 25 °C as for aging assays, except paraquat was added to NGM medium to 10 mm final concentration (ChemService, West Chester, PA, USA). For hydrogen peroxide, we scored 5-h survival of adult day 5 *fem-1(hc17)* animals in S-basal medium with indicated concentrations of hydrogen peroxide.

To determine lipofuscin levels, adult hermaphrodites were anesthetized in 0.2% sodium azide and mounted on 2% agarose pads for visualization of intestinal fluorescence on a Nikon E800 microscope using an Endow GFP filter with a mercury UV source (Nikon). Images were captured using a constant exposure time with a Hamamatsu ORCA digital CCD camera (Hamamatsu, Bridgewater, NJ, USA) using OpenLab software (Improvision, Lexingon, MA, USA). Lipofuscin levels were measured using ImageJ software (NIH Image) by determining average pixel intensity in each animal's intestine.

To measure levels of 4-HNE, animals were collected, fixed with 4% formaldehyde and permeabilized by digestion with type IV collagenase (Sigma Chemical Company), as described (Loer & Kenyon, 1993). Fixed and permeabilized specimens were incubated with anti4-HNE antisera (1 : 100 dilution, Genox Corp, Baltimore, MD, USA) for 2 h at 24 °C, washed and incubated overnight at 4 °C with Alexafluor-546-conjugated goat anti-mouse secondary antibody (1 : 100) (#A-11003, Invitrogen, Carlsbad, CA, USA). Stained animals were mounted on 2% agarose pads and fluorescence visualized as for lipofuscin with appropriate filter sets. 4-HNE immunofluorescence was measured in the pharynx terminal bulb and somatic gonad using ImageJ software.

### Analysis of gene expression by reverse transcriptase PCR

For reverse transcriptase PCR (RT-PCR) analysis of gene expression, *fem-1(hc17)* animals were grown at an approximate density of 200 animals/plate in the presence or absence of 200 µg mL^−1^ BB polyphenols. Animals were kept at 25 °C except for heat-stressed samples, which were transferred to 35 °C for 2 h before collection and processing. Animals were washed from plates with cold M9 buffer into Eppendorf tubes, and allowed to settle on ice. The worm pellet was resuspended in 300 µL Absolutely RNA lysis buffer (Stratagene, La Jolla, CA, USA) and stored frozen at −80 °C. RNA was prepared as per kit instructions. Complementary DNA was prepared with the ProStar Ultra HF RT-PCR kit (Stratagene). Real-time PCR was performed in an MJ Research Opticon thermal cycler (BioRad, Hercules, CA, USA), using SybrGreen 2x master mix (Applied Biosystems, Foster City, CA, USA) with 1 µm primers and 0.5 µL cDNA in a 25-µL reaction volume using the following gene-specific primers (Hsu *et al*., 2003): actin (*T04C12.6*), GTGTGACGACGAGGTTGCCGCTCTTGTTGTAGAC (F) and GGTAAGGATCTTCATGAGGTAATCAGTAAGATCAC (R); *hsp-12.6(F38E11.2)*, ATGATGAGCGTTCCAGTGATGGCTGACG (F) and TTAATGCATTTTTCTTGCTTCAATGTGAAGAATTCC (R); *hsp-16.1(T27E4.8)*, GTCACTTTACCACTATTTCCGTCCAGCTCATCAACGTTC (F) and CAACGGGCGCTTGCTGAATTGGAATAGATCTTCC (R); *hsp-16.49(Y46H3 A2)*, GCTCATGCTCCGTTCTCCATATTCTGATTCAAATGC (F) and GCAACAAAATTGATCGGAATAGAACGTGATGAG (R); and *hsp-70(F44E5.4)*, CGTTTCGAAGAACTGTGTGCTGATCTATTCCGG (F) and TTAATCA ACTTCCTCAACAGTAGGTCCTTGTGG (R).

## Supplementary material

The following tables and figure are available as supplementary material from:

**Table S1** Transcripts examined by RT-PCR in BB-treated animals

**Table S2** *hsp* induction in BB-treated animals vs. untreated controls at adult days 4 and 8 is in BB-treated animals

**Fig. S1** Beneficial effects of blueberry polyhenols did not require the activity of DAF-16/FOXO, which acts in the insulin-like signaling pathway in *Caenorhabditis elegans*. (A) *Lifespan of daf-16(mgDf50)* animals, completely lacking *daf-16* activity, was extended by treatment with blueberry polyphenols at indicated doses. (B) Blueberry polyphenols increased thermotolerance in *daf-16(mgDf50)* animals. Thermotolerance was assayed as for Fig. 5(A).

## References

[b1] Adachi H, Ishii N (2000). Effects of tocotrienols on life span and protein carbonylation in Caenorhabditis elegans. J. Gerontol. A Biol. Sci. Med. Sci.

[b2] An J, Blackwell T (2003). SKN-1 links C. elegans mesodermal specification to a conserved oxidative stress response. Genes Dev.

[b3] Ayyadevara S, Engle MR, Singh SP, Dandapat A, Lichti CF, Benes H, Shmookler Reis RJ, Liebau E, Zimniak P (2005). Lifespan and stress resistance of Caenorhabditis elegans are increased by expression of glutathione transferases capable of metabolizing the lipid peroxidation product 4-hydroxynonenal. Aging Cell.

[b4] Bolanowski M, Russell R, Jacobson L (1981). Quantitative measures of aging in the nematode Caenorhabditis elegans. I. Population and longitudinal studies of two behavioral parameters. Mech. Ageing Dev.

[b5] Brenner S (1974). The genetics of Caenorhabditis elegans. Genetics.

[b6] Brunk U, Terman A (2002). Lipofuscin: mechanisms of age-related accumulation and influence on cell function. Free Radic. Biol. Med.

[b7] Chiarpotto E, Biasi F, Scavazza A, Camandola S, Dianzani M, Poli G (1995). Metabolism of 4-hydroxy-2-nonenal and aging. Biochem. Biophys. Res. Commun.

[b8] Finkel T, Holbrook NJ (2000). Oxidants, oxidative stress and the biology of ageing. Nature.

[b9] Garigan D, Hsu A-L, Graser A, Kamath R, Ahringer J, Kenyon C (2002). Genetic analysis of tissue aging in Caenorhabditis elegans: a role for heat-shock factor and bacterial proliferation. Genetics.

[b10] Gems D, Riddle D (2000). Genetic, behavioral and environmental determinants of male longevity in Caenorhabditis elegans. Genetics.

[b11] Gems D, Sutton AJ, Sundermeyer ML, Albert PS, King KV, Edgley ML, Larsen PL, Riddle DL (1998). Two pleiotropic classes of daf-2 mutations affect larval arrest, adult behavior, reproduction and longevity in Caenorhabditis elegans. Genetics.

[b12] Glenn CF, Chow DK, David L, Cooke CA, Gami MS, Iser WB, Hanselman KB, Goldberg IG, Wolkow CA (2004). Behavioral deficits during early stages of aging in Caenorhabditis elegans result from locomotory deficits possibly linked to muscle frailty. J. Gerontol. A Biol. Sci. Med. Sci.

[b13] Golden T, Melov S (2004). Microarray analysis of gene expression with age in individual nematodes. Aging Cell.

[b14] Guarente L, Kenyon C (2000). Genetic pathways that regulate ageing in model organisms. Nature.

[b15] Harrington L, Harley C (1988). Effect of vitamin E on lifespan and reproduction in Caenorhabditis elegans. Mech. Ageing Dev.

[b16] Herndon LA, Schmeissner PJ, Dudaronek JM, Brown PA, Listner KM, Sakano Y, Paupard MC, Hall DH, Driscoll M (2002). Stochastic and genetic factors influence tissue-specific decline in ageing C. elegans. Nature.

[b17] Hosokawa H, Ishii N, Ishida H, Ichimori K, Nakazawa H, Suzuki K (1994). Rapid accumulation of fluorescent material with aging in an oxygen-sensitive mutant mev-1 of Caenorhabditis elegans. Mech. Ageing Dev.

[b18] Hsu AL, Murphy CT, Kenyon C (2003). Regulation of aging and age-related disease by DAF-16 and heat-shock factor. Science.

[b19] Huang C, Xiong C, Kornfeld K (2004). Measurements of age-related changes of physiological processes that predict lifespan of Caenorhabditis elegans. Proc. Natl Acad. Sci. USA.

[b20] Ishii N, Fujii M, Hartman PS, Tsuda M, Yasuda K, Senoo-Matsuda N, Yanase S, Ayusawa D, Suzuki K (1998). A mutation in succinate dehydrogenase cytochrome b causes oxidative stress and ageing in nematodes. Nature.

[b21] Joseph J, Shukitt-Hale B, Denisova N, Bielinski D, Martin A, McEwen J, Bickford P (1999). Reversals of age-related declines in neuronal signal transduction, cognitive, and motor behavioral deficits with blueberry, spinach, or strawberry dietary supplementation. J. Neurosci.

[b22] Joseph J, Shukitt-Hale B, Casadesus G (2005). Reversing the deleterious effects of aging on neuronal communications and behavior: beneficial properties of fruit polyphenolic compounds. Am. J. Clin. Nutr.

[b23] Kenyon C, Chang J, Gensch E, Rudner A, Tabtiang R (1993). A C. elegans mutant that lives twice as long as wild type. Nature.

[b24] Kim D, Feinbaum R, Alloing G, Emerson F, Garsin D, Inoue H, Tanaka-Hino M, Hisamoto N, Matsumoto K, Tan M-W, Ausubel F (2002). A conserved p38 MAP kinase pathway in Caenorhabditis elegans innate immunity. Science.

[b25] Kondo M, Yanase S, Ishii T, Hartman PS, Matsumoto K, Ishii N (2005). The p38 signal transduction pathway participates in the oxidative stress-mediated translocation of DAF-16 to Caenorhabditis elegans nuclei. Mech. Ageing Dev.

[b26] Lakowski B, Hekimi S (1998). The genetics of caloric restriction in Caenorhabditis elegans. Proc. Natl Acad. Sci. USA.

[b27] Larsen PL (1993). Aging and resistance to oxidative damage in Caenorhabditis elegans. Proc. Natl Acad. Sci. USA.

[b28] Lee S, Kennedy S, Tolonen A, Ruvkun G (2003). DAF-16 target genes that control C. elegans life-span and metabolism. Science.

[b29] Lithgow G, White T, Melov S, Johnson T (1995). Thermotolerance and extended life-span conferred by single-gene mutations and induced by thermal stress. Proc. Natl Acad. Sci. USA.

[b30] Liu R (2003). Health benefits of fruit and vegetables are from additive and synergistic combinations of phytochemicals. Am. J. Clin. Nutr.

[b31] Loer C, Kenyon C (1993). Serotonin-deficient mutants and male mating behavior in the nematode Caenorhabditis elegans. J. Neurosci.

[b32] Lund J, Tedesco P, Duke K, Wang J, Kim S, Johnson T (2002). Transcriptional profile of aging in *C. elegans*.. Curr. Biol.

[b33] Muñoz M, Riddle D (2003). Positive selection of Caenorhabditis elegans mutants with increased stress resistance and longevity. Genetics.

[b34] Murphy C, McCarroll S, Bargmann C, Fraser A, Kamath R, Li H, Kenyon C (2003). Genes that act downstream of DAF-16 to influence the lifespan of Caenorhabditis elegans. Nature.

[b35] Senoo-Matsuda N, Yasuda K, Tsuda M, Ohkubo T, Yoshimura S, Nakazawa H, Hartman PS, Ishii N (2001). A defect in the cytochrome b large subunit in complex II causes both superoxide anion overproduction and abnormal energy metabolism in Caenorhabditis elegans. J. Biol. Chem.

[b36] Solomon A, Bandhakavi S, Jabbar S, Shah R, Beitel G, Morimoto R (2004). *Caenorhabditis elegans* OSR-1 regulates behavioral and physiological responses to hyperosmotic environments. Genetics.

[b37] Tissenbaum H, Guarente L (2001). Increased dosage of a sir-2 gene extends lifespan in *Caenorhabditis elegans*. Nature.

[b38] Winkel-Shirley B (2002). Biosynthesis of flavonoids and effects of stress. Curr. Opin. Plant Biol.

[b39] Wood J, Rogina B, Lavu S, Howitz K, Helfand S, Tatar M, Sinclair D (2004). Sirtuin activators mimic caloric restriction and delay aging in metazoans. Nature.

[b40] Wu Z, Smith J, Paramasivam V, Butko P, Khan I, Cypser J, Luo Y (2002). Ginko biloba extract EGb 761 increases stress resistance and extends life span of Caenorhabditis elegans. Cell. Mol. Biol.

[b41] Youdim K, Shukitt-Hale B, MacKinnon S, Kalt W, Joseph J (2000). Polyphenolics enhance red blood cell resistance to oxidative stress: in vitro and in vivo. Biochim. Biophys. Acta.

[b42] Zheng W, Wang S (2003). Oxygen radical absorbing capacity of phenolics in blueberries, cranberries, chokeberries, and lingonberries. J. Agric. Food Chem.

